# 

**Published:** 1882-02

**Authors:** 


### CONTENTS:

ART. I.—The Chemistry and Physiological Action of Mercury as Used in Amalgam Filling. By E. S. Talbott, M D., D D. S ....................433

ART. II.—Pyorrhœa Alveolaris. By A. O. Rawls, D. D. S....................446

ART. III.—The Teeth of the Rising Generation. By C. E. Kells, D. D. S....................452

ART. IV—Civilization in its Relation to the Increasing Degeneracy of the Human Teeth. By Norman Kingsley, D D. S....................458

ART. V.—Tongue Sucking as a Cause of the Protrusion of the Incisor Teeth. By Appleby King, D. D. S....................462

ART. VI.—Hardening and Tempering Instruments. By Chas. A. Blake....................466

ART. VII.—Different Forms of Atrophy of the Teeth. By M. Parrot....................468

Forty-Second Annual Commencement of the Baltimore College of Dental Surgery....................470

### EDITORIAL, ETC.

Durability of Gutta Percha Fillings....................471
Discontinuance of the Dental Miscellany....................471

### OBITUARY.

Dr. Joshua Tucker. Resolutions on the Death of....................472
Dr. M. S. Dean....................473
Mr. Chas Abbey....................473

### BIBLIOGRAPHICAL.

The Opium Habit and Alcoholism....................473
The New England Journal of Dentistry....................474
The Prevention of Syphilis....................474
Dictionary Holder Noyes'....................474
Gratuitous Dentistry in the French Schools....................475
Transactions of Illinois State Dental Society....................475

### MONTHLY SUMMARY.

Uniting Gold to Amalgam in Tooth Filling....................475
Use of Platinum in Amalgam Alloys....................477
Catching Cold—Remedies....................478
Oxygenation of Amalgam....................480
Tempering Steel....................480

